# Evaluating the efficacy of adjuvant chemotherapy in cT1b-T2 patients with incidentally discovered positive lymph nodes after esophagectomy for esophageal squamous cell carcinoma: a retrospective cohort study

**DOI:** 10.1186/s12885-025-14472-7

**Published:** 2025-07-01

**Authors:** Hai-Bo Sun, Shao-Kang Feng, Xian-Ben Liu, Wen-Qun Xing, Pei-Nan Chen, Duo Jiang, Yang Liu

**Affiliations:** 1 Department of Thoracic Surgery, The Affiliated Cancer Hospital of Zhengzhou University (Henan Cancer Hospital), Zhengzhou City, Henan Province China; 2 Department of Thoracic Surgery, Anyang Tumor Hospital, Anyang City, Henan Province China; 3 Department of Radiation Oncology, The Affiliated Cancer Hospital of Zhengzhou University (Henan Cancer Hospital), No. 127 Dongming Road, Zhengzhou, 450008 Henan Province China

**Keywords:** Adjuvant chemotherapy, Esophageal squamous cell carcinoma, Lymph node, Survival, Esophagectomy

## Abstract

**Background:**

This study evaluated the association between adjuvant chemotherapy and the survival of cT1b-T2 patients with incidentally discovered positive lymph nodes (cN- but pN+) after esophagectomy for esophageal squamous cell carcinoma.

**Materials and methods:**

Esophageal cancer patients in whom positive lymph nodes were incidentally discovered after esophagectomy were enrolled in this retrospective cohort study. Patients were divided into the surgery alone and adjuvant chemotherapy groups. Propensity score matching (1:1) was used to minimize baseline differences.

**Results:**

In total, data from 343 patients who were incidentally discovered to have positive lymph nodes after surgery were analyzed. Each group consisted of 107 patients, with no significant difference in the background information between the two groups. There was also no significant difference in the overall survival (*P* = 0.227) or disease-free survival (*P* = 0.210) between the groups in the matched overall study population. Notably, in subgroup analysis, The disease-free survival in the adjuvant chemotherapy group was significantly better than that in the operation group alone for patients with pathological stage T3 (*P* = 0.023). Multivariate analysis showed that male (ref: female, HR = 1.796, 95% CI, 1.013–3.183, *P* = 0.045) was a significant independent predictive factor for overall survival.

**Conclusion:**

Adjuvant chemotherapy may improve survival for patients with cT1b-T2 but pathological T3 stage patients with incidentally discovered node-positive esophageal squamous cell carcinoma.

## Introduction

Esophageal cancer is the ninth most common malignancy worldwide, and squamous cell carcinoma accounts for approximately 90% of incident esophageal cancers worldwide [1–3]. Central Asia and East Asia are the areas with the highest incidence of esophageal squamous cell carcinoma (ESCC) in the world; in particular, in China, the morbidity and mortality of esophageal cancer are among the top five, and the pathological type of the majority of patients is squamous cell carcinoma [4–7]. 

Esophagectomy is the standard treatment for patients with resectable esophageal cancer, but the 5-year overall survival (OS) rate after surgery is not satisfactory. Multidisciplinary combined therapy has benefitted patients with locally advanced resectable esophageal carcinoma [8–10]. However, a phase III multicenter randomized trial (FFCD9901) showed that neoadjuvant radiotherapy and chemotherapy did not improve the R0 resection rate or survival in patients with clinical stage I and II esophageal carcinoma compared with surgery alone [11]. For patients with cT1b-T2N0 ESCC, the National Comprehensive Cancer Network (NCCN) clinical practice guidelines recommend direct esophagectomy and surveillance after R0 (cancer-free resection margins) resection, regardless of postoperative lymph node (LN) status [12]. However, current NCCN recommendations for the postoperative management of esophageal cancer lack support from relevant randomized trials. Although several clinical trials have shown that postoperative adjuvant chemotherapy is beneficial for patients with ESCC [13], given the changes to the perioperative treatment guidelines, a better understanding of which patients actually benefit from postoperative treatment is needed. Currently, the survival benefit of adjuvant chemotherapy (AC) for patients with cT1b-T2N0 ESCC presenting with incidentally discovered positive lymph nodes (LNs) is still unclear.

The purpose of this study was to compare the survival effects of AC on patients with cT1b-T2 ESCC presenting with incidentally discovered positive LNs compared with those of surgery alone (SA) and to identify predictive factors that influence survival.

## Patients and methods

### Patient population

**Fig. 1:**
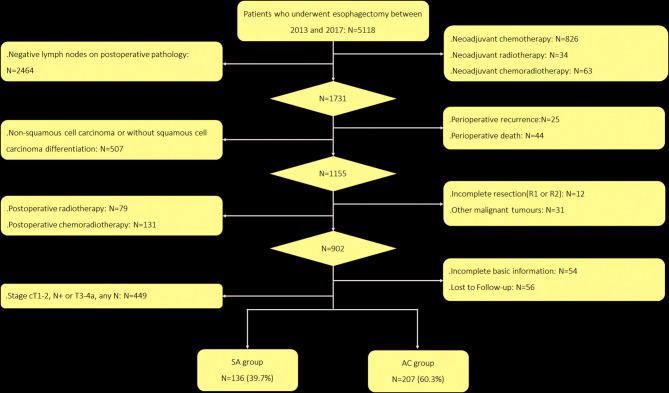
Study flow diagram

Between January 1, 2013, and December 12, 2017, 5118 patients who underwent esophagectomy were recruited from the Affiliated Cancer Hospital of Zhengzhou University (Henan Cancer Hospital). This study was approved by the Review Ethics Board of The Affiliated Cancer Hospital of Zhengzhou University (Henan Cancer Hospital), and consent was obtained from all the participants. The following patients were excluded from the analysis: (1) those who accepted neoadjuvant chemoradiotherapy, neoadjuvant chemotherapy, and neoadjuvant radiotherapy (*n* = 923); (2) those in whom a positive lymph node (LN) was not found in postoperative pathology (*n* = 2464); (3) those who died or developed metastasis within 60 days after surgery (*n* = 69); (4) those with nonsquamous cell carcinoma or without squamous cell carcinoma differentiation (*n* = 507); (5) those who underwent incomplete resection (R1 or R2) or with malignant tumors in other organs (*n* = 43); (6) those who received radiotherapy or chemoradiotherapy after surgery (*n* = 210); (7) those with incomplete basic information or were lost to follow-up (either at the first follow-up within three months after surgery or two consecutive follow-ups within six months after the first loss) (*n* = 110); and (8) patients with stage cT1-T2, N + or T3-T4a, any N disease (*n* = 449). The remaining 343 patients with stage cT1-2 N0 but pN + disease were divided into the SA group and AC group. Since there is no uniform postoperative treatment strategy, whether the patient received chemotherapy after surgery was at the discretion of the surgeon. All patients in the AC group started chemotherapy within 40 days after surgery and completed at least one cycle of chemotherapy, and patients who did not complete one cycle of chemotherapy were included in the SA group. The flow chart of patient screening is shown in Fig. 1.

### Data collection

Clinical staging was based on patient history; physical examination; pulmonary-function tests; electrocardiogram (ECG); routine hematologic and biochemical tests; endoscopic ultrasonography with biopsies; upper gastrointestinal contrast; cardiac and cervical ultrasonography; computed tomography (CT) scans of the brain, thorax, and abdomen; and bone scintigraphy. Positron emission tomography (PET) with fluorodeoxyglucose is used when distant metastasis is suspected. All patients in this study underwent the same preoperative examinations. cN status was determined by endoscopic ultrasonography (EUS), neck ultrasound, computed tomography (CT) of the chest and abdomen and positron emission tomography (PET) with fluorodeoxyglucose. When all preoperative examinations did not report lymph node metastasis, and the short diameter of mediastinal and abdominal lymph nodes was < 10 mm, it was considered cN-. Patients with stage cN- ESCC who are pN + are regarded as having incidentally discovered nodal positivity. The Charlson Comorbidity Index and Clavien-Dindo grade were used to describe preoperative comorbidities and postoperative complications, respectively [14, 15]. Perioperative blood transfusion was any transfusion in the period from 60 days before surgery to 60 days after surgery. Patients in the tumor length ≤ 3 cm group were considered low risk. The degree of tumor differentiation, tumor location, nerve invasion, lymphovascular invasion and TNM stage were based on the eighth edition of the American Joint Committee on Cancer (AJCC) TNM staging system [16]. All esophagectomy procedures included minimally invasive McKeown esophagectomy, open McKeown esophagectomy, left thoracotomy with cervical or chest anastomosis, and Ivor-Lewis gastric tube reconstruction of the digestive tract.

### Postoperative chemotherapy regimens

In the current study, a total of 207 patients (60.3%) received postoperative adjuvant chemotherapy. Among these patients, approximately 59.9% (124 of 207) of patients received paclitaxel and platinum chemotherapy regimen (paclitaxel: 135–175 mg/m^2^, intravenously, day 1, and cisplatin: 20–25 mg/m^2^, intravenously, days 2–4, every 3 weeks), 22.7% (47 of 207) of patients received fluorouracil and platinum chemotherapy regimen (capecitabine: 1000 mg/m^2^, per oral, days 1–14, and oxaliplatin: 130 mg/m^2^, intravenously, day 1, every 3 weeks), 5.8% (12 of 207) of patients received fluorouracil, platinum and paclitaxel chemotherapy regimen (5-fluorouracil: 750 mg/m^2^, intravenously, days 1–5, and oxaliplatin, 80 mg/m^2^, intravenously, day 1, and docetaxel, 70 mg/m^2^, intravenously, day 1), 2.9% (6 of 207) of patients received fluorouracil single-agent chemotherapy (capecitabine: 1000–1250 mg/m^2^, twice a day, per oral, days 1–14, every 3 weeks, or 5-fluorouracil: 800 mg/m^2^, continue 24 h, intravenously, days 1–5, every 4 weeks), 1% (2 of 207) of patients received fluorouracil and paclitaxel chemotherapy regimen (5-fluorouracil: 1800 mg/m^2^, continue 72 h, intravenously, and paclitaxel: 175 mg/m^2^, intravenously, day 1, every 4 weeks), 3.9% (8 of 207) patients received Irinotecan-based or vincristine-based chemotherapy regimens, and The remaining 3.9% (8 of 207) patients received other regimens. Chemotherapy regimens changed over time.

### Follow-up of survival and recurrence

For asymptomatic patients after surgery, chest and abdominal computed tomography (CT) and abdominal and cervical ultrasound examinations were performed every three months for 1–2 years, and regular examinations were performed every six months in years 3 to 5 and once yearly after 5 years. During follow-up, esophageal dilatation under gastroscopy was used to relieve the symptoms of anastomotic stricture after esophagectomy. Needle biopsy, positron emission tomography (PET) with fluorodeoxyglucose, emission computed tomography (ECT) and magnetic resonance imaging (MRI) were optionally performed if recurrence was suspected. Metastasis of LNs or solid organs confirmed by an imaging examination or needle biopsy was regarded as recurrence. OS and disease-free survival (DFS) were calculated from the date of surgery. The end of follow-up was August 16, 2020.

### Statistical analysis

All continuous variables are described as the median and interquartile range, and categorical variables are described as percentages. The Mann-Whitney U test was used to compare differences in continuous variables, and the chi-square test or Fisher’s exact test was used to compare differences in categorical variables. We performed propensity score matching analysis to alleviate selection and allocation biases between the two groups. Age, sex, Charlson comorbidity score, tumor location, tumor length, number of dissected LNs, number of positive LNs, number of LN dissection stations, number of positive LN stations, nerve invasion, lymphovascular invasion, histology, pathological differentiation grade, pathological T stage, pathological N stage, TNM stage, surgical approach, thoracic duct ligation, perioperative blood transfusion and Clavien-Dindo grade were included in the logistic regression curve to calculate the propensity score. The matching caliper was set at 0.1, and the SA group and AC group were matched one to one. Kaplan-Meier survival curves were used to calculate OS and DFS, and the log-rank test was used for comparisons. A Cox proportional hazards regression model was used to identify the factors that independently affected the survival of patients with ESCC. Some continuous variables in the Cox proportional hazards regression model (such as age, number of dissected LNs, number of positive LNs, number of LN stations dissected, and number of positive LN stations) were grouped by the median as the cutoff point. In the multivariate analysis of the Cox proportional hazards regression model, only variables with *P* < 0.2 in the univariate analysis were introduced. All tests were double-sided, and *P* < 0.05 was considered statistically significant. SPSS 23 (SPSS Inc., Chicago, IL) was used for all social science statistics.

### Characteristics

A total of 343 patients were enrolled in the present study. No patients had pathological stage N3 disease, 255 (74.3%) patients had pathological stage N1 disease, and 88 (25.7%) patients had pathological stage N2 disease. Before matching, 136 (39.7%) patients underwent SA, and 207 (60.3%) patients received at least one cycle of AC after surgery. In the AC group, the patients were younger (*P* < 0.001) and had more number of positive LNs (*P* = 0.025), fewer LN stations dissected (*P* = 0.011), poorly differentiated tumors (*P* = 0.007), a higher proportion of pathological stage N2 disease (*P* = 0.025), and less perioperative blood transfusion (*P* = 0.032) (Table 1). After propensity score matching, 107 patients in the SA group and 107 patients in the AC group were included in the analysis. The baseline characteristics of the patients were well balanced between the groups (Table 1).

**Table 1:** Comparison of the baseline characteristics between groups

Variable	Overall population *n* = 343 (100%)	Before matching		*P*-value	After matching		*P*-value
		SA group *n* = 136 (39.7%)	AC group *n* = 207 (60.3%)		SA group *n* = 107 (50%)	AC group *n* = 107 (50%)	
Age, years (IQR)	63 (58–68)	65 (61–70)	61 (57–66)	< 0.001	62 (58–67)	64 (60–69)	0.055
Sex				0.074			0.885
Male	231 (67.3%)	84 (61.8%)	147 (71.0%)		71 (66.4%)	70 (65.4%)	
Female	112 (32.7%)	52 (38.2%)	60 (29.0%)		36 (33.6%)	37 (34.6%)	
Charlson comorbidity score				0.232			0.884
0	266 (77.6%)	100 (73.5%)	166 (80.2%)		82 (76.6%)	85 (79.4%)	
1	51 (14.9%)	22 (16.2%)	29 (14.0%)		16 (15.0%)	14 (13.1%)	
≥ 2	26 (7.6%)	14 (10.3%)	12 (5.8%)		9 (8.4%)	8 (7.5%)	
Tumor location				0.411			0.512
Upper	32 (9.3%)	16 (11.8%)	16 (7.7%)		13 (12.1%)	8 (7.5%)	
Middle	117 (34.1%)	47 (34.6%)	70 (33.8%)		37 (34.6%)	38 (35.5%)	
Lower	194 (56.6%)	73 (53.7%)	121 (58.5%)		57 (53.3%)	61 (57.0%)	
Tumor length(cm)				0.904			1.000
≤ 3	155 (45.2%)	62 (45.6%)	93 (44.9%)		54 (50.5%)	54 (50.5%)	
> 3	188 (54.8%)	74 (54.4%)	114 (55.1%)		53 (49.5%)	53 (49.5%)	
No. of resected LNs (IQR)	22 (15–30)	23 (15–31)	21 (15–29)	0.269	22 (15–30)	23 (14–28)	0.777
No. of positive LNs (IQR)	1 (1–3)	1 (1–3)	2 (1–3)	0.025	1 (1–2)	1 (1–2)	0.339
No. of resected LN stations (IQR)	6 (5–7)	6 (5–8)	6 (5–7)	0.011	6 (5–8)	6 (5–7)	0.274
No. of positive LN stations (IQR)	1 (1–2)	1 (1–2)	1 (1–2)	0.084	1 (1–2)	1 (1–2)	0.366
Nerve invasion				0.570			1.000
Yes	15 (4.4%)	7 (5.1%)	8 (3.9%)		3 (2.8%)	4 (3.7%)	
No	328 (95.6%)	129 (94.9%)	199 (96.1%)		104 (97.2%)	103 (96.3%)	
Lymphovascular invasion				0.375			0.344
Yes	66 (19.2%)	23 (16.9%)	43 (20.8%)		19 (17.8%)	14 (13.1%)	
No	277 (80.8%)	113 (83.1%)	164 (79.2%)		88 (82.2%)	93 (86.9%)	
Histology				0.170			0.172
SCC	297 (86.6%)	122 (89.7%)	175 (84.5%)		95 (88.8%)	88 (82.2%)	
SCC with other differentiation	46 (13.4%)	14 (10.3%)	32 (15.5%)		12 (11.2%)	19 (17.8%)	
Pathological differentiation grade				0.007			0.271
Well differentiated	18 (5.2%)	13 (9.6%)	5 (2.4%)		7 (6.5%)	2 (1.9%)	
Moderately differentiated	157 (45.8%)	65 (47.8%)	92 (44.4%)		50 (46.7%)	52 (48.6%)	
Poorly differentiated	168 (49.0%)	58 (42.6%)	110 (53.1%)		50 (46.7%)	53 (49.5%)	
Pathological T^a^ stage				0.373			0.077
T1	61 (17.8%)	24 (17.6%)	37 (17.9%)		24 (22.4%)	13 (12.1%)	
T2	185 (53.9%)	68 (50.0%)	117 (56.5%)		61 (57.0%)	62 (57.9%)	
T3	97 (28.3%)	44 (32.4%)	53 (25.6%)		22 (20.6%)	32 (29.9%)	
Pathological N^a^ stage				0.025			0.385
N1	255 (74.3%)	110 (80.9%)	145 (70.0%)		89 (83.2%)	84 (78.5%)	
N2	88 (25.7%)	26 (19.1%)	62 (30.0%)		18 (16.8%)	23 (21.5%)	
TNM^a^ stage				0.708			0.125
IIB	43 (12.5%)	17 (12.5%)	26 (12.6%)		17 (15.9%)	9 (8.4%)	
IIIA	155 (45.2%)	65 (47.8%)	90 (43.5%)		58 (54.2%)	55 (51.4%)	
IIIB	145 (42.3%)	54 (39.7%)	91 (44.0%)		32 (29.9%)	43 (40.2%)	
Surgical approach				0.310			0.266
MIE	132 (38.5%)	59 (43.4%)	73 (35.3%)		47 (43.9%)	41 (38.3%)	
Left thoracotomy and cervical anastomosis	119 (34.7%)	45 (33.1%)	74 (35.7%)		35 (32.7%)	37 (34.6%)	
McKeown	73 (21.3%)	28 (20.6%)	45 (21.7%)		23 (21.5%)	20 (18.7%)	
Left thoracotomy and chest anastomosis	17 (5.0%)	4 (2.9%)	13 (6.3%)		2 (1.9%)	7 (6.5%)	
Ivor-Lewis	2 (0.6%)	0 (0.0%)	2 (1.0%)		0 (0.0%)	2 (1.9%)	
Thoracic duct ligation				0.782			0.784
Yes	157 (45.8%)	61 (44.9%)	96 (46.4%)		54 (50.5%)	56 (52.3%)	
No	186 (54.2%)	75 (55.1%)	111 (53.6%)		53 (49.5%)	51 (47.7%)	
Perioperative blood transfusion				0.032			1.000
Yes	9 (2.6%)	7 (5.1%)	2 (1.0%)		2 (1.9%)	2 (1.9%)	
No	334 (97.4%)	129 (94.9%)	205 (99.0%)		105 (98.1%)	105 (98.1%)	
Clavien-Dindo grade				0.054			0.053
Grade 0	130 (37.9%)	58 (42.6%)	72 (34.8%)		48 (44.9%)	39 (36.4%)	
Grade 1–2	138 (40.2%)	44 (32.4%)	94 (45.4%)		32 (29.9%)	49 (45.8%)	
Grade 3a-4b	75 (21.9%)	24 (25.0%)	41 (19.8%)		27 (25.2%)	19 (17.8%)	

Comparison of baseline characteristics in the SA group and the AC group (matched and unmatched)

*AC*, adjuvant chemotherapy; *IQR*, interquartile range; *LN*, lymph node; *MIE*, minimally invasive esophagectomy; *SA*, surgery alone; *SCC*, squamous cell carcinoma

^a^According to the 8th edition of the AJCC staging system

Two hundred and seven patients received AC. The majority of chemotherapy regimens employed were on the paclitaxel-platinum platform or fluorouracil-platinum platform (Table 2). One hundred twenty-four (59.9%) patients received paclitaxel/platinum combination chemotherapy regimens, and 47 (22.7%) patients received fluorouracil/platinum combination chemotherapy regimens.

**Table 2:** Postoperative chemotherapy regimens

Chemotherapeutic Agent	AC group *n* = 207 (100%)
Paclitaxel/Platinum Combo	124 (59.9%)
Fluorouracil/Platinum Combo (without Paclitaxel)	47 (22.7%)
Fluorouracil/Platinum/Paclitaxel Combo	12 (5.8%)
Fluorouracil (without Platinum and Paclitaxel)	6 (2.9%)
Fluorouracil/Paclitaxel Combo (without Platinum)	2 (1.0%)
Other	8 (3.9%)
Unknown	8 (3.9%)

*All chemotherapy regimens used in the AC group. The majority of patients received chemotherapy based on paclitaxel/platinum or fluorouracil/platinum*

*AC*, adjuvant chemotherapy

### Univariate and multivariate survival analyses

In the univariate survival analysis, variables with *p* < 0.2 were included in the multivariate Cox proportional hazards model (Table 3). In the unmatched cohort, these variables included AC, sex, Charlson Comorbidity Index, tumor length, number of resected LN stations, number of positive LN stations, pathological T stage, TNM stage, surgical approach, and thoracic duct ligation. In the multivariate survival analysis, receiving AC was independently associated with improved OS (hazard ratio (HR) = 0.569, 95% CI, 0.390–0.830, *P* = 0.003). In addition, male (ref: female, HR = 1.884, 95% CI, 1.197–2.841, *P* = 0.006), Charlson comorbidity score 1 (ref: 0, HR = 0.531, 95% CI, 0.293–0.960, *P* = 0.036), number of positive LN stations (ref: <2, HR = 1.651, 95% CI, 1.095–2.491, *P* = 0.017), pathological T2 stage (ref: T1, HR = 3.046, 95% CI, 0.909–10.202, *P* = 0.071) and pathological T3 stage (ref: T1, HR = 5.207, 95% CI, 1.322–20.513, *P* = 0.018) were independent prognostic factors related to OS. After matching, the univariate survival analysis indicated that AC was not an independent prognostic factor associated with OS (HR = 0.750, 95% CI, 0.468–1.201, *P* = 0.231). Sex, the Charlson Comorbidity Index, tumor length, number of resected LNs, number of resected LN stations, pathological T stage, TNM stage, surgical approach, and thoracic duct ligation were included in the multivariate Cox proportional hazards model. In the multivariate survival analysis, male (ref: female, HR = 1.796, 95% CI, 1.013–3.183, *P* = 0.045) was found to be an independent factor for OS.

**Table 3:** Univariate and multivariate analyses of the predictors of Disease-Free survival

Variable	Before matching				After matching			
	Univariate analysis		Multivariate analysis		Univariate analysis		Multivariate analysis	
	HR (95% CI)	*P*-value	HR (95% CI)	*P*-value	HR (95% CI)	*P*-value	HR (95% CI)	*P*-value
AC (ref: No)								
Yes	0.717 (0.499–1.028)	0.070	0.569 (0.390–0.830)	0.003	0.750 (0.468–1.201)	0.231		
Age, years (ref: ≤63)								
>63	1.013 (0.707–1.452)	0.942			1.341 (0.837–2.150)	0.222		
Sex (ref: Female)								
Male	1.508 (0.998–2.280)	0.051	1.844 (1.197–2.841)	0.006	1.707 (0.988–2.948)	0.055	1.796 (1.013–3.183)	0.045
Charlson comorbidity score (ref: 0)								
1	0.659 (0.369–1.177)	0.159	0.531 (0.293–0.960)	0.036	0.548 (0.236–1.270)	0.161	0.598 (0.252–1.418)	0.243
≥ 2	1.227 (0.657–2.293)	0.521	1.456 (0.761–2.786)	0.256	1.156 (0.527–2.534)	0.718	1.154 (0.515–2.584)	0.728
Tumor location (ref: Upper)								
Middle	0.969 (0.494–1.901)	0.928			1.087 (0.462–2.560)	0.849		
Lower	1.186 (0.628–2.239)	0.598			1.311 (0.588–2.921)	0.508		
Tumor length (ref: ≤3)								
> 3	1.628 (1.121–2.366)	0.011	1.116 (0.748–1.665)	0.590	1.857 (1.150–2.999)	0.011	1.208(0.727–2.009)	0.465
No. of resected LNs (ref: ≤22)								
>22	0.814 (0.562–1.179)	0.276			0.671 (0.414–1.086)	0.104	1.081 (0.609–1.920)	0.790
No. of positive LNs (ref: <2)								
≥2	1.227 (0.856–1.758)	0.226			0.933 (0.578–1.507)	0.778		
No. of resected LN stations (ref: ≤6)								
>6	0.645 (0.433–0.959)	0.030	0.730 (0.467–1.143)	0.169	0.403 (0.231–0.705)	0.001	0.574 (0.288–1.145)	0.115
No. of positive LN stations (ref: <2)								
≥ 2	1.588 (1.104–2.285)	0.013	1.651 (1.095–2.491)	0.017	0.935 (0.541–1.617)	0.811		
Nerve invasion (ref: No)								
Yes	0.848 (0.312-2.300)	0.745			0.889 (0.218–3.634)	0.870		
Lymphovascular invasion (ref: No)								
Yes	0.843 (0.515–1.379)	0.496			1.052 (0.537–2.061)	0.883		
Histology (ref: SCC with other differentiation)								
SCC	1.069 (0.631–1.811)	0.904			1.098 (0.561–2.150)	0.785		
Pathological differentiation grade (ref: Poorly differentiated)								
Moderately/Well differentiated	0.909 (0.635-1.300)	0.601			0.790 (0.494–1.264)	0.326		
Pathological T^a^ stage (ref: T1)								
T2	2.560 (1.270–5.159)	0.009	3.046 (0.909–10.202)	0.071	4.445 (1.370-14.419)	0.013	3.899 (0.517–29.381)	0.187
T3	5.712 (2.805–11.631)	< 0.001	5.207(1.322–20.513)	0.018	11.373 (3.460-37.382)	< 0.001	5.798 (0.623–53.929)	0.122
Pathological N^a^ stage (ref: N1)								
N2	1.162 (0.778–1.736)	0.463			0.904 (0.485–1.684)	0.751		
TNM^a^ Stage (ref: IIB)								
IIIA	2.102 (0.897–4.923)	0.087	0.588 (0.141–2.448)	0.465	3.645 (0.874–15.212)	0.076	0.715 (0.064–7.993)	0.785
IIIB	4.689 (2.033–10.816)	< 0.001	0.814 (0.167–3.974)	0.799	8.934 (2.146–37.190)	0.003	1.186 (0.092–15.235)	0.896
Surgical approach (ref: MIE)								
OE	1.459 (0.993–2.145)	0.054	1.095 (0.675–1.777)	0.713	1.726 (1.047–2.845)	0.032	1.031 (0.539–1.975)	0.926
Thoracic duct ligation (ref: No)								
Yes	1.586 (1.097–2.294)	0.014	1.082 (0.692–1.692)	0.730	1.824 (1.129–2.946)	0.014	1.118 (0.617–2.026)	0.714
Perioperative blood transfusion (ref: No)								
Yes	0.525 (0.130–2.127)	0.367			0.515 (0.071–3.715)	0.510		
Clavien-Dindo grade (ref: Grade 0)								
Grade 1–2	0.795 (0.530–1.192)	0.267			0.760 (0.437–1.321)	0.330		
Grade 3a-4b	0.810 (0.502–1.306)	0.387			1.127 (0.629–2.020)	0.688		

*Predictors of overall survival using Cox proportional hazards regression*

*AC*, adjuvant chemotherapy; *CI*, confidence interval; *HR*, hazard ratio; *LN*, lymph node; *MIE*, minimally invasive esophagectomy; *OE*, open esophagectomy; *SCC*, squamous cell carcinoma

^a^According to the 8th edition of the AJCC staging system

### OS analyses

**Fig. 2:**
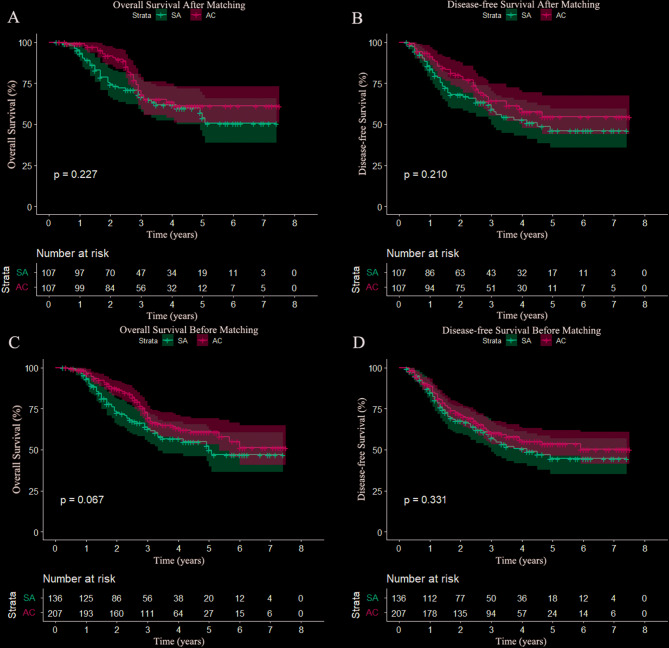
Overall survival and disease-free survival curves. **(A)** Overall survival among matched patients (*p* = 0.227). **(B)** Disease-free survival among matched patients (*p* = 0.210). **(C)** Overall survival among unmatched patients (*p* = 0.067). **(D)** Disease-free survival among unmatched patients (*p* = 0.311)

The median follow-up time was 47.0 months. In the unmatched cohort, the 3-year OS rates of the SA group and the AC group were 62.8% and 69.4%, and the 5-year OS rates were 46.9% and 58.0% (*P* = 0.067)(Fig. 2C)., respectively. In the matched groups, the 3-year OS rates of the SA group and the AC group were 66.6% and 67.9%, and the 5-year OS rates were 50.5% and NA, respectively (*P* = 0.227)(Fig. 2A). No significant difference was found between the two groups before and after matching.

### DFS analyses and metastasis

Before matching, the 3-year DFS rates of the SA group and AC group were 57.2% and 60.1%, and the 5-year DFS rates were 44.6% and 50.2%, respectively (*P* = 0.331)(Fig. 2D). After matching, the 3-year DFS rates of the SA group and AC group were 59.1% and 61.3%, and the 5-year DFS rates were 46.1% and NA, respectively (*P* = 0.210) (Fig. 2B). The numbers of metastases and recurrences in the SA group (75/143, 52.4%) were higher than those in the AC group (68/143, 47.6%) (Table 4). Esophagus or mediastinum (74/143,51.7%) and lung or pleura (24/143,16.8%) were more easily to develop metastasis and recurrence.

**Table 4:** Metastasis or recurrence site

Metastasis or Recurrence Site	SA group *n* = 75 (52.4%)	AC group *n* = 68 (47.6%)	Overall population *n* = 143 (100%)
Esophagus or Mediastinum	46 (61.3%)	28 (41.2%)	74 (51.7%)
Neck	4 (5.3%)	5 (7.4%)	9 (6.3%)
Lung or Pleura	11 (14.7%)	13 (19.1%)	24 (16.8%)
Liver	4 (5.3%)	6 (8.8%)	10 (7.0%)
Abdomen	3 (4.0%)	3 (4.4%)	6 (4.2%)
Bone	4 (5.3%)	7 (10.3%)	11 (7.7%)
Other	1 (1.3%)	4 (5.9%)	5 (3.5%)
Unknown	2 (2.7%)	2 (2.9%)	4 (2.8%)

*The sites of recurrence and metastasis in all patients. Recurrence and metastasis were more common in the esophagus and mediastinum (51.7%)*

*AC*, adjuvant chemotherapy; *SA*, surgery alone

### Subgroup analysis

**Fig. 3:**
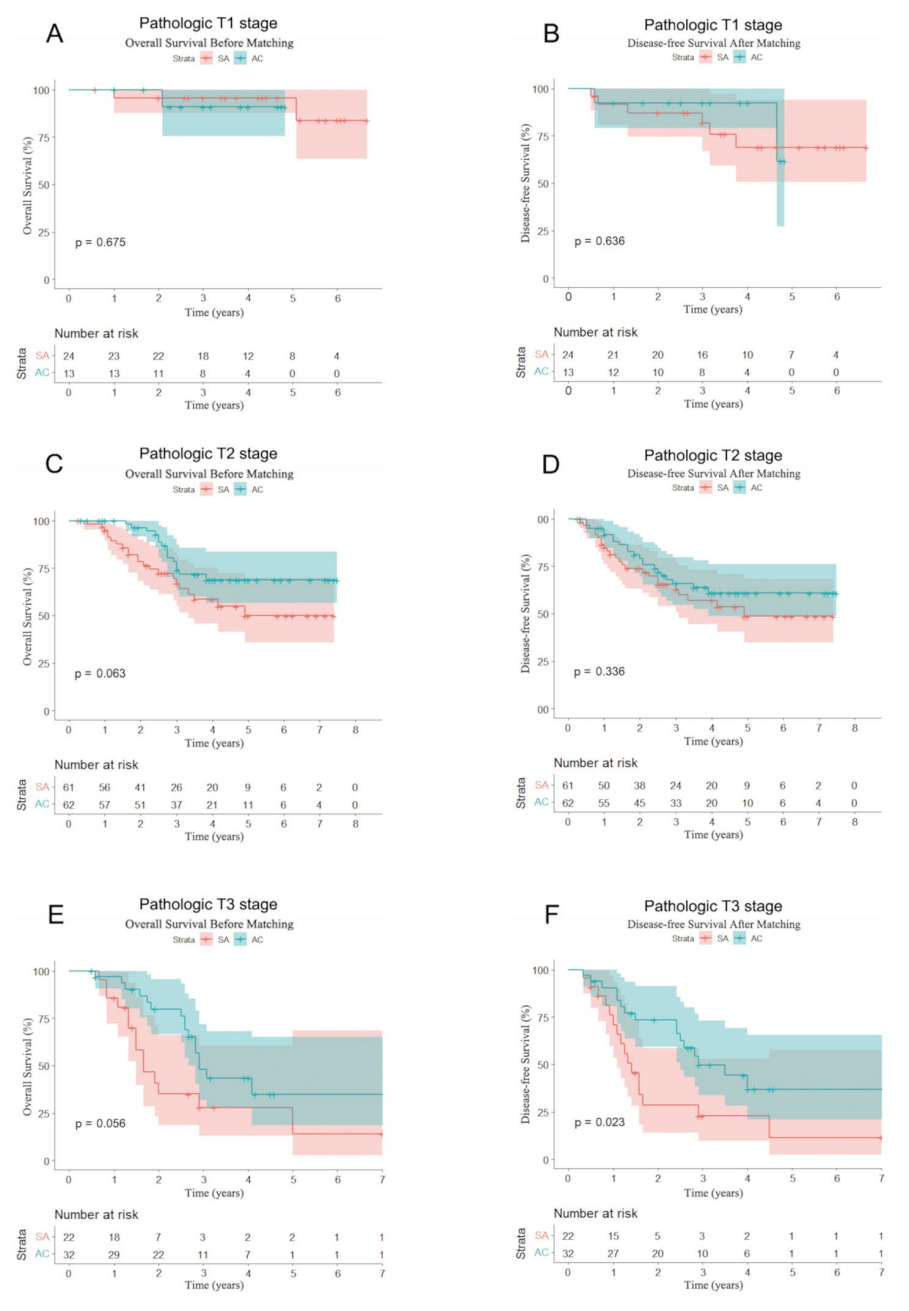
Overall survival and disease-free survival curves according to pathologic T stage in matched patients. **A** and **B**. Overall survival and Disease-free survival among matched patients with pathologic T1 stage. **C** and **D**. Overall survival among Disease-free survival matched patients pathologic T2 stage. **E** and **F**. Overall survival and Disease-free survival among matched patients pathologic T3 stage

We performed a subgroup analysis of matched patients based on pathological T staging. No significant difference in OS and DFS was observed for patients with pathological stage T1 after matching, the 5-year OS rates of the SA group and AC group were 83.7% and NA (*P* = 0.675), and the 5-year DFS rates were 68.9% and 61.5% (*P* = 0.636), respectively. For patients with pathological stage T2, after matching, the 5-year OS rates of the SA group and the AC group were 49.8% and 68.7% (*P* = 0.063), and the 5-year DFS rates were 48.6% and 60.9% (*P* = 0.336), respectively. From the survival curve, the OS in the AC group was better than that in the SA group, although neither OS nor DFS reached a statistical difference. For patients with pathological stage T3, after matching, the 5-year OS rates in the SA group and the AC group were 14.0% and 34.8% (*P* = 0.056), and the 5-year DFS rates were 11.4% and 36.9% (*P* = 0.023), respectively. From the survival curve, the OS and DFS in the AC group were significantly better than those in the SA group. And statistically, the DFS in the AC group was better than that in the SA group (Fig. 3).

## Discussion

Currently, the treatment strategy for patients with incidentally discovered node-positive ESCC after surgery is unclear. We retrospectively analyzed the effects of AC on patients with cT1b-T2 ESCC in whom positive LNs were incidentally discovered after R0 resection. Our study shows that AC does not provide a survival benefits for patients with incidentally discovered node-positive(cN- but pN+) cT1b-T2 who do not receive induction therapy in the overall propensity score matching analysis. Notably, AC significantly improved DFS for patients with incidentally discovered node-positive(cN- but pN+) pathological T3 stage ESCC in subgroup analyses based on pathological T staging.

The prognosis of node-positive patients who are treated by SA is poor, and whether AC has a survival benefit for patients with cT1b-T2 ESCC presenting with incidentally discovered positive LNs (cN- but pN+) is still inconclusive. Several randomized controlled trials showed that postoperative AC did not improve OS in patients with LN-positive ESCC [17–19]. Only JCOG9204, a phase III study performed by Ando et al., concluded that postoperative AC with two courses of cisplatin and fluorouracil could prolong DFS for patients with node-positive esophageal cancer compared with SA in the pathological N1 subgroup (*p* = 0.041) [18]. Several other retrospective studies have concluded that AC can improve outcomes for patients with ESCC [20–22]. However, based on the current NCCN guidelines, some patients in the abovementioned studies may have received a greater survival benefit from induction therapy than from postoperative AC [23]. Therefore, the point of extrapolation of the data from these studies to support the use of AC for patients with incidentally discovered node-positive cT1b-T2N0 ESCC is unclear. At present, the NCCN guidelines recommend neoadjuvant chemoradiation or neoadjuvant chemotherapy followed by surgery for the primary treatment of cN + resectable ESCC [12]. Most patients with locally advanced resectable esophageal cancer receive induction therapy. Patients with stage cT1b-T2 disease were the primary source of patients with incidentally discovered positive LNs (cN- but pN+) who did not receive induction therapy. Our study were no patients with pathological stage N3 disease, and the results showed that AC improved survival in patients with incidentally discovered node-positive(cN- but pN+) cT1b-T2 but pathological T3 stage ESCC. Although there have been numerous reports on the role of AC in node-positive ESCC, few studies have investigated the effect of AC on survival in patients with incidentally discovered node-positive cT1b-T2 ESCC. Gao et al. conducted a retrospective analysis comparing the outcomes of surgical intervention alone, AC, and adjuvant chemoradiation therapy among patients with cT1-2 N0 disease discovered to be pathologically node positive [13]. They found that AC may be sufficient for margin-negative patients among those with cT1-2 N0 esophageal cancer and incidental pN + disease. However, there was no significant improvement in survival for patients treated with AC in our overall study population after matching. This may be related to the fact that only patients with ESCC were included in our study. After all, esophageal adenocarcinomas did benefit from AC in previous studies [10]. Of note, in subgroup analysis, patients with pathological T3 stage had a survival benefit from AC. This is consistent with the findings of Jeon, YJ et al. [24] They retrospectively reviewed the survival outcomes of node-positive patients with ESCC who underwent curative resection with or without AC, and the results showed that AC might improved survival in patients with node-positive ESCC, especially those with pathological T3-4 stage. The NCCN guidelines recommend regular follow-up for patients with node-negative ESCC stage after direct surgery, and for patients with adenocarcinoma, regular follow-up or chemoradiotherapy or chemotherapy may be considered. Considering the inconsistencies in the biological characteristics of esophageal adenocarcinoma and ESCC, as well as recent findings, whether patients with incidentally discovered node-positive(cN- but pN+) ESCC need AC may require grouping based on high-risk factors, especially pathological T stage.

To date, the survival benefit of AC for cT1b-T2 ESCC patients with incidentally discovered positive LNs (cN- but pN+) is still unclear. Because neoadjuvant chemotherapy or neoadjuvant chemoradiation has been shown to improve the survival rate of patients with resectable ESCC presenting with positive LNs, we believe that patients with esophageal cancer should undergo a complete preoperative examination and that stricter tumor staging is very important. Despite advances in staging techniques, clinical staging is not very reliable, and the number of false-negative LNs remains significant. Among patients with stage cT1–2, N0 disease, approximately 30% still have LN metastasis after surgery [25, 26]. Patients with undetected LN metastasis may continue to receive surgery as the main method of treatment. Increasing the diagnostic rate of positive LNs appears to indirectly improve survival in patients with incidentally discovered positive LNs (cN- but pN+). The combination of positron emission tomography/computed tomography (PET/CT), MRI and endoscopic ultrasound (EUS) has been shown to improve the preoperative staging accuracy of tumors [27–29]. Improved preoperative staging has an advantage over AC in improving the survival of ESCC patients who are incidentally discovered to have positive LNs.

This study has several limitations. First, like all retrospective studies, our results are subject to selection bias, although we strived to reduce this bias through propensity score matching. Second, chemotherapy regimens are not uniform, and different chemotherapy regimens may affect our results. Third, due to the limited number of patients from a single center, it is difficult to draw definitive conclusions about the optimal treatment for patients who are incidentally discovered to have positive LNs after surgery. Fourth, although we recorded the basic information of patients to the greatest extent possible, we did not examine the side effects of chemotherapy. Fifth, the accuracy of preoperative staging may have affected our research.

## Conclusions

AC does provide a survival benefit for patients with cT1b-T2 but pathological T3 stage ESCC with incidentally discovered positive LNs (cN- but pN+). Further prospective studies are needed to confirm the results.

## Data Availability

Data were available on reasonable request from the the corresponding author.

## Abbreviations

AC: Adjuvant chemotherapy
CI: Confidence interval
DFS: Disease-free survival
ESCC: Esophageal squamous cell carcinoma
HR: Hazard ratio
LN: Lymph node
LNs: Lymph nodes
OS: Overall survival
SA: Surgery alone
